# CLEC4E-Mediated Macrophage Activation Contributes to Host Defense in A*canthamoeba* Keratitis

**DOI:** 10.1167/iovs.67.12.63

**Published:** 2026-10-26

**Authors:** Qingquan Shi, Zhen Cheng, Zhenyu Wei, Jinding Pang, Mingda Wei, Zijun Zhang, Sitong Shen, Boling Xu, Qingfeng Liang

**Affiliations:** 1 Beijing Institute of Ophthalmology, Beijing Tongren Eye Center, Beijing Tongren Hospital, Capital Medical University, Beijing, China

**Keywords:** *Acanthamoeba* keratitis, CLEC4E, macrophage, HIF-1α, pattern recognition receptors

## Abstract

**Purpose:**

*Acanthamoeba* keratitis (AK) is a vision-threatening corneal infection characterized by intense inflammation and tissue damage. Although innate immunity is critical for host defense against *Acanthamoeba*, the pattern recognition receptors (PRRs) involved in pathogen sensing remain incompletely defined. This study investigated the role of the C-type lectin receptor CLEC4E in macrophage responses during AK.

**Methods:**

Transcriptomic data from human AK corneas were analyzed to identify immune-related pathways and PRRs. RAW264.7 macrophages and bone marrow–derived macrophages were infected with *Acanthamoeba* trophozoites and subjected to RNA sequencing, quantitative real-time PCR, and Western blot analyses. CLEC4E function was examined using the agonist trehalose-6,6′-dibehenate and lentiviral short hairpin RNA–mediated knockdown. An experimental mouse model of AK was used to assess the effects of in vivo CLEC4E blockade.

**Results:**

A transcriptomic analysis of AK corneas revealed enhanced inflammatory signaling and increased expression of multiple PRRs, including CLEC4E. *Acanthamoeba* infection induced rapid and sustained CLEC4E upregulation in macrophages, accompanied by activation of nuclear factor κB signaling and increased expression of inflammatory mediators. Pharmacological activation of CLEC4E amplified inflammatory responses, whereas CLEC4E knockdown reduced cytokine production and nuclear factor κB activation. Correlation and functional analyses identified hypoxia-inducible factor 1-α (HIF-1α) as a mediator associated with CLEC4E-dependent inflammatory amplification. Inhibition of HIF-1α attenuated CLEC4E-enhanced inflammatory responses. In vivo, the neutralization of CLEC4E aggravated experimental AK, resulting in increased corneal opacity and clinical severity.

**Conclusions:**

CLEC4E promotes macrophage activation and contributes to protective innate immune responses during *Acanthamoeba* infection. HIF-1α mediates CLEC4E-dependent inflammatory amplification, highlighting a potential link between CLEC4E signaling and macrophage inflammatory programs during AK.

*A* *canthamoeba* keratitis (AK) is a severe and potentially vision-threatening corneal infection caused by free-living protozoa of the genus *Acanthamoeba.*^1^^,^^2^ The incidence of AK has increased worldwide in recent decades, largely associated with the widespread use of contact lenses.^3^^–^^5^ Clinically, AK is characterized by progressive corneal inflammation and can lead to significant visual impairment or even blindness.^6^^–^^8^ Although antiamoebic therapies are available, treatment outcomes remain inconsistent due to delayed diagnosis and the persistence of amoebae within corneal tissue.^9^^–^^11^ Increasing evidence suggests that host immune responses play a critical role in controlling *Acanthamoeba* infection and shaping disease progression.^12^^–^^14^ However, the mechanisms by which the innate immune system recognizes *Acanthamoeba* remain incompletely understood.

Innate immune recognition represents a critical first line of defense in the cornea and is largely mediated by pattern recognition receptors (PRRs), which detect conserved pathogen-associated molecular patterns and initiate antimicrobial responses. Previous studies have shown that certain PRRs, particularly Toll-like receptors (TLRs) such as TLR2 and TLR4, participate in host responses during *Acanthamoeba* infection.^15^^–^^17^ Nevertheless, these findings provide only a partial view of PRR-mediated immunity in AK. The broader repertoire of PRRs involved in this disease remains incompletely defined, especially the potential contribution of C-type lectin receptors (CLRs), a major class of innate immune receptors best known for their roles in antifungal and antibacterial host defense.^18^

Among the PRRs expressed by macrophages, CLRs have emerged as important sensors of microbial pathogens. CLEC4E (macrophage-inducible C-type lectin, Mincle) recognizes microbial glycolipids and damage-associated molecular patterns and promotes macrophage activation and antimicrobial immunity.^18^^–^^20^ However, whether CLEC4E contributes to macrophage responses during *Acanthamoeba* infection remains unknown, and its contribution to macrophage activation and inflammatory responses during *Acanthamoeba* infection has not been defined.

In this study, we analyzed transcriptomic profiles of corneal tissues from patients with AK and observed enhanced innate immune signaling accompanied by an increased expression of multiple PRRs. Using an in vitro macrophage infection model, we further investigated the role of CLEC4E in macrophage responses to *Acanthamoeba*. Our findings suggest that the CLEC4E–hypoxia-inducible factor 1-α (HIF-1α) axis contributes to macrophage activation and may represent a previously unrecognized pathway involved in host defense against AK.

## Methods

### Human Corneal Transcriptome Data Acquisition

Human corneal RNA sequencing data were retrieved from the cohort previously reported by Wei et al. (2023).^21^ Briefly, corneal tissues were collected from three patients (two females and one male) with advanced-stage AK undergoing corneal transplantation and three donors from the Beijing Tongren Eye Bank. The AK diagnosis was based on typical clinical manifestations and laboratory confirmation of *Acanthamoeba* infection. Patients with mixed ocular infections, ocular inflammation, ocular trauma, ocular surgery history, cancer, or chronic infections were excluded. The mean age of AK patients and controls was 44.0 ± 17.0 and 46.0 ± 13.6 years (*P* = 0.903), respectively.

For transcriptomic analysis, one-half of each corneal button was used for RNA extraction. Total RNA was extracted using TRIzol reagent, and RNA quality was assessed using NanoDrop and an Agilent Bioanalyzer (Agilent Technologies, Santa Clara, CA, USA) (RNA Integrity Number > 7.0). Poly-A mRNA was purified from 1 µg total RNA for library construction. Sequencing was performed using the Illumina NovaSeq 6000 platform (Illumina, San Diego, CA, USA) with paired-end 150-bp sequencing. Raw sequencing reads were filtered using fastp and aligned to the human reference genome (GRCh38) using HISAT2. Transcript assembly and quantification were performed using StringTie, and gene expression levels were calculated as fragments per kilobase of transcript per million mapped reads.

Differential expression analysis was performed using DESeq2 based on raw read counts. Genes with |log2 fold change| ≥ 0.5 and adjusted *P* values of <0.05 were considered differentially expressed. Multiple testing correction was performed using the Benjamini–Hochberg false discovery rate method. Gene Set Enrichment Analysis was performed using MSigDB gene sets, including Hallmark gene sets, with significance determined according to the normalized enrichment score (|NES| > 1) and an adjusted *P* value of <0.05. Nonmetric multidimensional scaling analysis was conducted using Bray–Curtis dissimilarity. Gene Ontology (GO) and Kyoto Encyclopedia of Genes and Genomes (KEGG) pathway enrichment analyses were conducted using clusterProfiler.

### Cell Culture

The murine macrophage cell line RAW264.7 was obtained from Wuhan Pricella Biotechnology Co., Ltd. (Wuhan, China) and cultured in DMEM (Gibco, Grand Island, NY, USA) supplemented with 10% fetal bovine serum (Gibco) and 1% penicillin–streptomycin (Gibco). Cells were maintained at 37°C in a humidified 5% CO₂ atmosphere.

Bone marrow–derived macrophages (BMDMs) were generated from C57BL/6 mice. Briefly, bone marrow cells were flushed from the femurs and tibias and cultured in DMEM supplemented with 10% fetal bovine serum and 20 ng/mL macrophage colony-stimulating factor (GenScript Biotech Corporation, Nanjing, China). After 7 days of culture, differentiated macrophages were harvested for subsequent experiments.

### *Acanthamoeba* Culture and Infection

*Acanthamoeba* trophozoites were cultured in PYG medium at 28°C. Before infection, cells were washed with PBS and replaced with fresh RPMI 1640 medium without antibiotics. Logarithmic-phase trophozoites were harvested, washed with sterile PBS, and added to macrophage cultures at a multiplicity of infection of 1:5. Cells were harvested at 2 and 6 hours post infection for downstream analyses. Control groups received equal volumes of PBS.

### RNA Extraction and RNA Sequencing of Macrophages

Total RNA was extracted using TRIzol Reagent according to the manufacturer's instructions. RNA quality was assessed using an Agilent 5300 Bioanalyzer, and concentration was measured using a NanoDrop ND-2000 spectrophotometer. Only high-quality RNA samples meeting the following criteria were used for library construction: concentration, ≥20 ng/µL; total RNA, >1 µg; and RNA Integrity Number, >6.5.

RNA purification, reverse transcription, library construction, and sequencing were performed by Shanghai Majorbio Bio-pharm Biotechnology Co., Ltd. (Shanghai, China). For conventional input samples, libraries were prepared using the Illumina Stranded mRNA Prep, Ligation kit (Illumina) with 1 µg total RNA. Briefly, poly(A) mRNA was enriched using oligo(dT) beads, fragmented. Double-stranded cDNA was synthesized using random hexamer primers, followed by end-repair, phosphorylation, adapter ligation, size selection (300–400 bp), and PCR amplification (10–15 cycles). Libraries were quantified using Qubit 4.0 and sequenced on the Illumina NovaSeq X Plus platform in paired-end 150-bp mode.

Raw paired-end reads were quality trimmed using fastp with default parameters. Clean reads were aligned to the mouse reference genome (GRCm39) using HISAT2. Transcript assembly was performed using StringTie in a reference-based approach. Gene expression levels were quantified as transcripts per million using RSEM. Differential expression analysis was performed using DESeq2 based on the raw expected counts derived from RSEM. Differentially expressed genes were identified using the thresholds of |log2 fold change| ≥ 0.5 and a false discovery rate of <0.05. GO functional enrichment analysis and KEGG pathway analysis were performed using Goatools and custom Python scripts utilizing the scipy library, respectively. A Bonferroni-corrected *P* value of <0.05 was considered statistically significant.

### CLEC4E Activation and HIF-1α Inhibition

For CLEC4E activation, macrophages were pretreated with 10 µg/mL trehalose-6,6′-dibehenate (TDB; Aladdin Shanghai Biochemical Technology Co., Shanghai, China) for 24 hours before infection. After pretreatment, cells were washed and infected in fresh RPMI 1640 medium without continued TDB exposure. For HIF-1α inhibition, macrophages were pretreated with 10 µM YC-1 (MedChemExpress, Monmouth Junction, NJ, USA) for 1 hour before infection. During infection, YC-1 was maintained in the culture medium to ensure sustained inhibition. Control groups received equivalent volumes of DMSO.

### Lentiviral Short Hairpin RNA (shRNA) Knockdown of Clec4e

Three lentiviral vectors encoding shRNA targeting mouse Clec4e and a nontargeting negative control were constructed and packaged by Sangon Biotech (Shanghai, China). RAW264.7 cells were seeded in 24-well plates and transduced with lentiviral particles at a multiplicity of infection of 80. After 24 hours, the medium was replaced with fresh complete medium. Forty-eight hours post transduction, cells were subjected to puromycin selection to establish stable knockdown lines. Knockdown efficiency was confirmed by quantitative real-time PCR and Western blot analysis before subsequent infection experiments.

### Establishment of the AK Model

Female C57BL/6 mice, aged 8 weeks, were acquired from Vital River Laboratory Animal Technology Co., Ltd. (Beijing, China), and allowed 1 week to adapt in a specific pathogen-free animal room. All animal procedures were performed in accordance with the ARVO Statement for the Use of Animals in Ophthalmic and Visual Research.

To establish the AK model, mice were anesthetized with 0.1% sodium pentobarbital (Sigma, St. Louis, MO, USA) via intraperitoneal injection. Subsequently, 1 µL of *Acanthamoeba* suspension containing 10⁴ trophozoites was injected into the corneal stroma of the left eye using a 33G syringe (Hamilton 21–2062). Only the left eyes were used for infection experiments.

After infection, mice were randomly allocated into experimental groups. To evaluate the clinical course of keratitis, a cohort of mice (*n* = 6 per group) was monitored by slit-lamp microscopy for ≤7 days post infection. Clinical severity was evaluated using a previously established scoring system based on three parameters: corneal opacity area, opacity density, and surface regularity. Each parameter was graded from 0 to 4 according to predefined criteria, and the total clinical score was calculated as the sum of the three parameters. Detailed scoring criteria are provided in Supplementary Table S1.^22^ To assess the validity of the composite score, we examined the intercorrelations among the three parameters using Spearman's rho and variance inflation factors. To minimize observer bias, slit-lamp examinations and clinical scoring were performed by an investigator who was blinded to the treatment allocation. Mice were coded with anonymous identification numbers by another investigator before clinical evaluation, and the groups were revealed only after the completion of data analysis.

For molecular analyses, a separate cohort of mice (*n* = 6 per group) was euthanized at day 1 post infection, and corneal tissues were collected for quantitative real-time PCR analysis. Furthermore, a third independent cohort of mice (*n* = 6 per group) was euthanized at day 3 post infection to quantify the *Acanthamoeba* burden.

For in vivo blockade of CLEC4E signaling, mice received a 2.5-µL subconjunctival injection of anti-CLEC4E (Mincle) monoclonal antibody (MBL, D292-3M2) 24 hours before *Acanthamoeba* inoculation. Control mice received equal amounts of rat IgG2b isotype control antibody (Sigma-Aldrich, M5409). This preinfection blockade strategy was designed to evaluate the contribution of CLEC4E signaling to the innate immune defense during the initial stage of infection.

### Measurement of the *Acanthamoeba* Burden

To examine the pathogen load, mice were euthanized and their corneas were harvested at day 3 post infection. Corneas were snap-frozen and homogenized using a tissue lyser (Ningbo Scientz Biotech Co., Ltd., Ningbo, China). Total DNA was extracted using the QIAamp DNA Mini Kit (Qiagen Sciences, Germantown, MD; Cat# 51304) according to the manufacturer's instructions. The parasitic burden was quantified by quantitative real-time PCR using primers and a probe specific for *Acanthamoeba* 18S rRNA (forward: 5′-CGACCAGCGATTAGGAGACG-3′; reverse: 5′-CCGACGCCAAGGACGAC-3′; probe: 5′-6FAM-TGAATACAAAACACCACCATCGGCGC-BHQ1-3′). The primer sequences and PCR protocol were adopted from a previous study.^23^

### Quantitative Real-Time PCR

Total RNA was extracted from cultured cells using the RNA Easy Fast Tissue/Cell Kit (Tiangen, Beijing, China). cDNA was synthesized using HiScript III All-in-One RT SuperMix (Vazyme, Nanjing, China). Quantitative real-time PCR was performed using the Taq Pro Universal SYBR qPCR Master Mix (Vazyme) on an ABI 7500 Real-Time PCR System (Applied Biosystems). The amplification protocol consisted of 95°C for 10 minutes; 40 cycles of 95°C for 15 seconds and 60°C for 60 seconds. Relative gene expression was calculated using the 2^−ΔΔCt method. Peptidylprolyl isomerase A was used as the endogenous reference gene. The primer sequences are listed in Supplementary Table S2.

### Western Blotting

Cells were lysed in RIPA buffer containing protease and phosphatase inhibitors. Equal amounts of protein were separated by SDS–PAGE and transferred to polyvinylidene fluoride membranes. Membranes were blocked with 5% nonfat milk and incubated overnight at 4°C with primary antibodies against inducible nitric oxide synthase (iNOS, 1:1000, ab178945, Abcam, Cambridge, UK), TNF-α (1:1000, ab183218, Abcam), IL-1β (1:1000, ab52915, Abcam), phosphorylated p65 (1:1000, ab76302, Abcam), total p65 (1:1000, ab76311, Abcam), CLEC4E (1:1000, PA5-116924, Invitrogen, Carlsbad, CA, USA), and β-tubulin (1:10000, ab6046, Abcam). After incubation with horseradish peroxidase–conjugated secondary antibodies, signals were detected using enhanced chemiluminescence.

### Statistical Analysis

All statistical analyses were performed using R software (version 4.4.2; The R Foundation for Statistical Computing, Vienna, Austria). Continuous variables are presented as mean ± SEM and were analyzed using Welch's *t*-test for two-group comparisons or one-way ANOVA followed by Tukey's post hoc test for multiple comparisons. Ordinal variables were analyzed using the Mann–Whitney *U* test for two-group comparisons. A two-sided *P* value of <0.05 was considered statistically significant.

## Results

### Enhanced Inflammatory and PRR Signatures in AK Corneas

In the previous study,^21^ the transcriptional landscape of late-stage human AK was established using bulk RNA sequencing. That initial analysis revealed 2668 differentially expressed genes (1477 upregulated and 1191 downregulated), identifying a global shift in the immune and metabolic pathways. Specifically, key immunomodulatory genes were prominently altered, including the significant upregulation of the M2-associated regulators SLAMF7 and STAT6. Pathway enrichment demonstrated widespread alterations in the chemokine signaling pathway, cell adhesion molecules, and cytokine–cytokine receptor interactions (Supplementary Tables S3 and S4). However, the broader repertoire of innate PRRs driving the initial inflammatory response remained uncharacterized. Building directly on this previously established dataset, the current study reanalyzed this dataset to specifically investigate the activation of innate immune-sensing mechanisms. A nonmetric multidimensional scaling analysis showed a clear separation between the AK and control samples, indicating distinct transcriptional profiles (Fig. 1A). Differential expression analysis further revealed extensive gene expression changes in AK corneas compared with controls (Fig. 1B). Among the upregulated genes, several immune-related molecules, including SIGLEC1, GBP5, and CD68, were identified, suggesting enhanced macrophage infiltration and activation in AK corneas.

**Figure 1.**
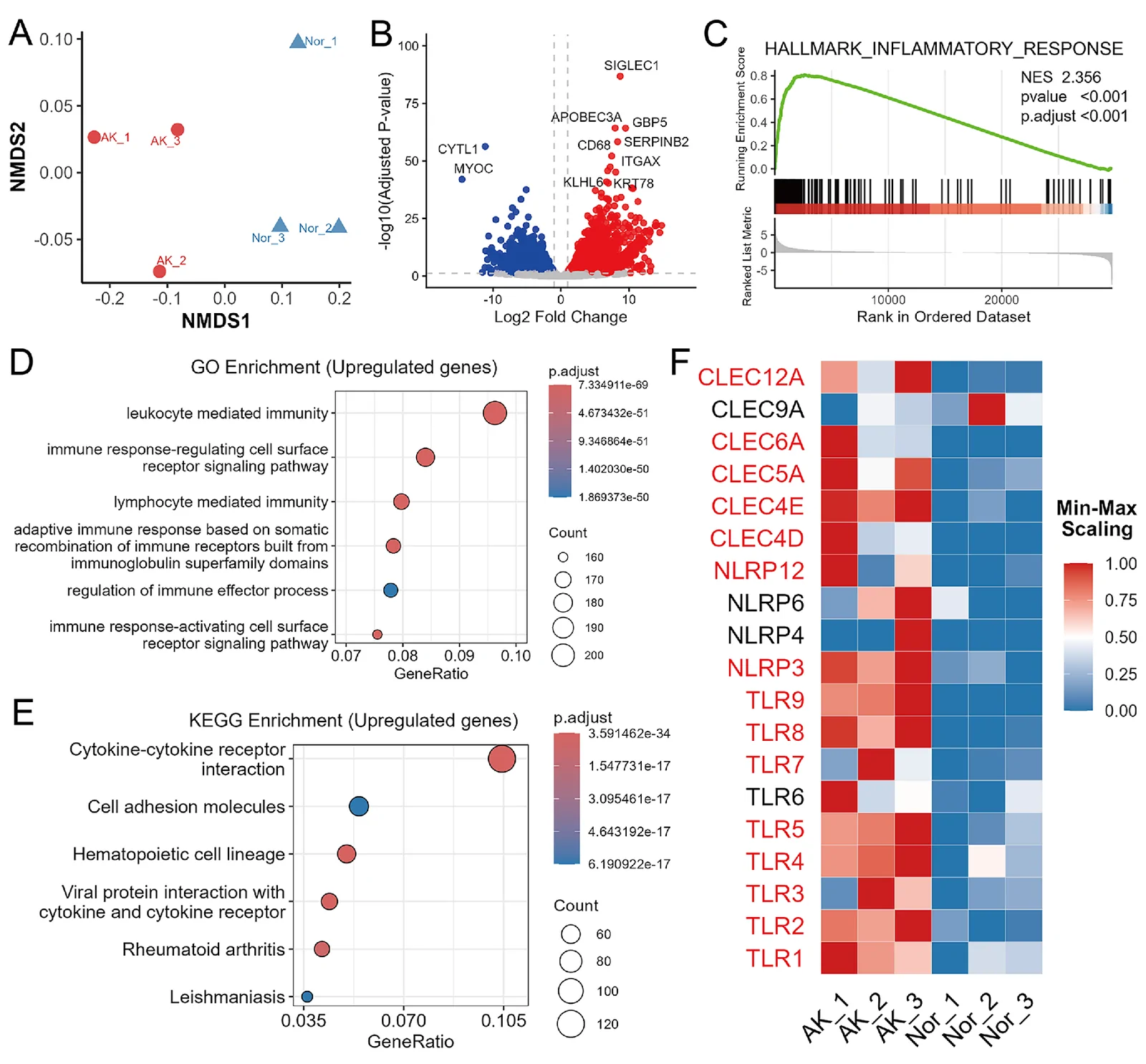
Enhanced inflammatory transcriptional programs and PRR signatures in the corneas of AK patients. (**A**) Nonmetric multidimensional scaling (NMDS) analysis in corneal tissues from AK patients and healthy controls. (**B**) Volcano plots showing differentially expressed genes between AK and control corneas. (**C**) Gene Set Enrichment Analysis (GSEA) of inflammatory response–related gene sets. (**D**) GO enrichment and (**E**) KEGG pathway analysis of upregulated genes in AK corneas. (**F**) Heatmap showing the expression of PRRs, including CLR, NLR, and TLR family members, in AK and control corneas. The human corneal transcriptomic data analyzed in this figure are derived from the cohort previously established and described by Wei et al.^21^

To further investigate pathway-level alterations, Gene Set Enrichment Analysis was conducted. Inflammatory response–related gene sets were significantly enriched in AK corneas (Fig. 1C), indicating the activation of inflammation-associated transcriptional programs. Consistently, GO enrichment analysis of upregulated genes revealed significant enrichment in leukocyte-mediated immunity, immune response–regulating cell surface receptor signaling pathways, and lymphocyte-mediated immunity (Fig. 1D). KEGG pathway analysis further identified enrichment in cytokine–cytokine receptor interaction, hematopoietic cell lineage, and infection-related pathways (Fig. 1E), highlighting broad immune activation within the AK cornea. To validate the robustness of our transcriptomic findings, we performed a sensitivity analysis on upregulated genes using a stricter threshold of a log2 fold change of ≥1.0. GO and KEGG pathway enrichment analyses under this threshold revealed consistent enrichment of key inflammatory signaling pathways in human corneas (Supplementary Fig. S1). Importantly, heatmap analysis demonstrated increased expression of multiple PRRs, including members of the TLR (TLR1, TLR2, TLR3, TLR4, TLR5, TLR7, TLR8, and TLR9), NLR (NLRP3 and NLRP12), and CLR (CLEC4D, CLEC4E, CLEC5A, CLEC6A, and CLEC12A) families, in AK corneas compared with controls (Fig. 1F). Collectively, these findings indicate that *Acanthamoeba* infection is associated with enhanced innate immune recognition and robust inflammatory signaling within corneal tissue.

### *Acanthamoeba* Infection Promotes Inflammatory and Clec4e-Associated Responses in Macrophages

Because corneal tissue contains diverse resident and infiltrating cell populations, we next established an in vitro macrophage infection model to determine whether *Acanthamoeba* directly induces PRR expression in macrophages. RAW264.7 cells were infected and harvested at 2 and 6 hours post infection for RNA sequencing. The 2-hour time point was selected to capture early transcriptional responses associated with initial pattern recognition signaling, whereas the 6-hour time point was intended to reflect subsequent transcriptional reprogramming, including responses driven by cytokine-mediated signaling. Four experimental groups were included: uninfected macrophages at 2 hours (M2), *Acanthamoeba*-infected macrophages at 2 hours (AM2), uninfected macrophages at 6 hours (M6), and *Acanthamoeba*-infected macrophages at 6 hours (AM6) (Fig. 2A). Nonmetric multidimensional scaling analysis revealed distinct clustering between the infected and control macrophages at both time points (Fig. 2B), indicating rapid transcriptional reprogramming. At 2 hours post infection, macrophages displayed a significant enrichment of inflammatory pathways, including MAPK, TNF, IL-17, and nuclear factor κB (NF-κB) signaling (Figs. 2C, 2D). Gene Set Enrichment Analysis confirmed significant enrichment of TNF-α and IL-1β response gene sets (Fig. 2E). Notably, multiple PRRs were upregulated at this early time point, including *Tlr2*, *Tlr4*, *Tlr7*, and *Nlrp3*, as well as the CLR family members *Clec4d*, *Clec4e*, and *Clec12a* (Fig. 2F), suggesting the activation of innate immune-sensing mechanisms.

**Figure 2.**
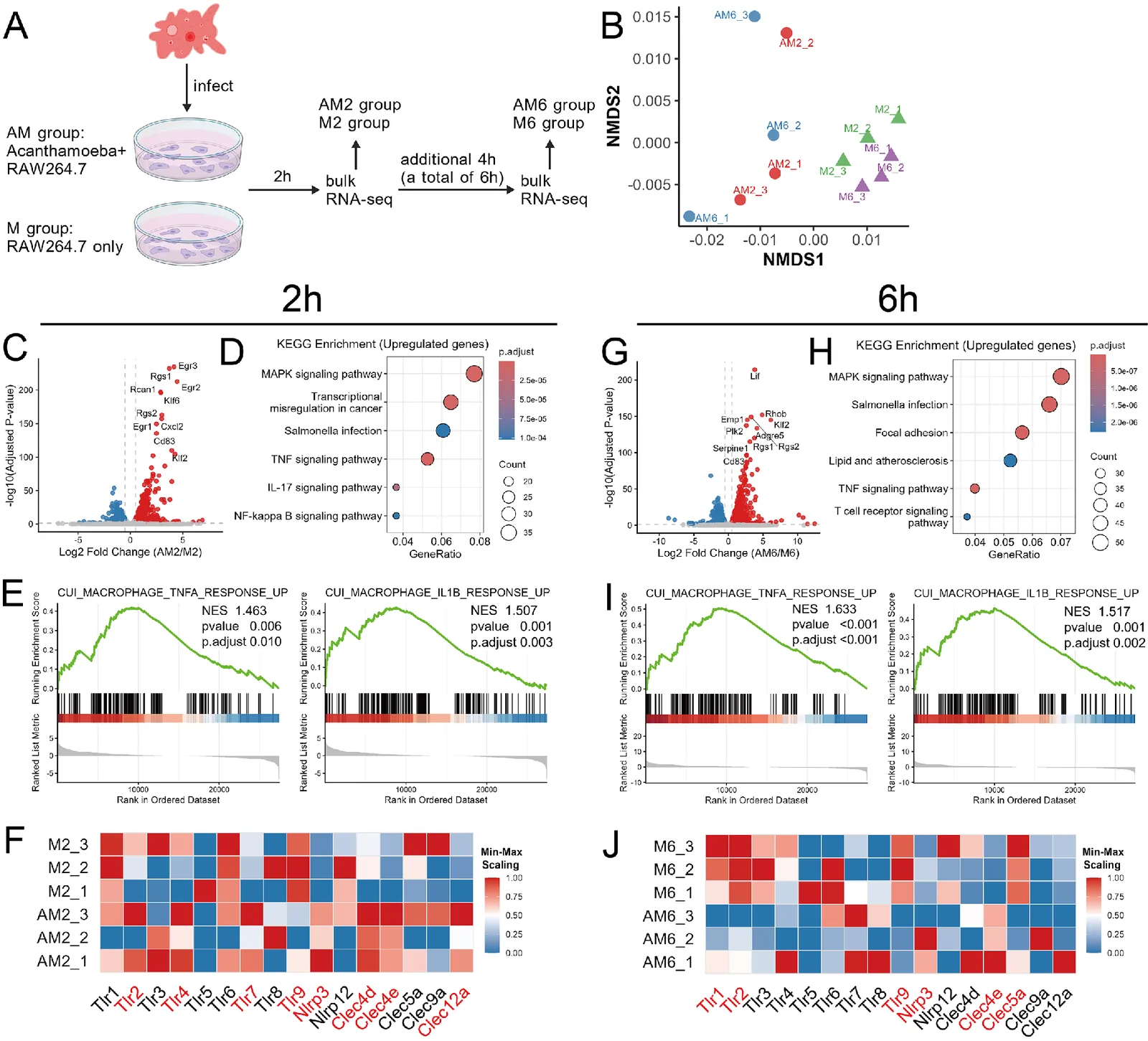
Transcriptomic profiling of macrophages following *Acanthamoeba* infection. (**A**) Experimental design of bulk RNA sequencing (RNA-seq) in RAW264.7 macrophages at 2 and 6 hours post infection. (**B**) A nonmetric multidimensional scaling (NMDS) analysis revealed distinct clustering between the infected and control macrophage samples. (**C**, **G**) Volcano plots showing differentially expressed genes between the AM2 and M2 groups (**C**) and between the AM6 and M6 groups (**G**). (**D**, **H**) KEGG pathway enrichment analysis of upregulated genes at (**D**) 2 hours post infection and (**H**) 6 hours post infection. (**E**, **I**) Gene Set Enrichment Analysis (GSEA) of TNF-α and IL-1β response gene sets at (**E**) 2 hours post infection and (**I**) 6 hours post infection. (**F**, **J**) Heatmaps showing expression of PRRs in RAW264.7 cells at (**F**) 2 hours post infection and (**J**) 6 hours post infection.

At 6 hours post infection, inflammatory pathway enrichment persisted and expanded to include T-cell receptor signaling and focal adhesion pathways (Figs. 2G, 2H). TNF-α and IL-1β response signatures remained significantly enriched (Fig. 2I). Among PRRs, Clec4e and Nlrp3 exhibited sustained and significant upregulation at both 2 and 6 hours (Fig. 2J). This temporal pattern of PRR expression suggests a transition from early pathogen recognition toward a later phase of inflammatory amplification, potentially involving increasing contributions from damage-associated signals as host cell injury accumulates. To ensure the robustness of these findings, a sensitivity KEGG enrichment was performed on upregulated genes using a stricter threshold of a log2 fold change of ≥1.0. Consistent with the primary analysis, core inflammatory signaling pathways—including the TNF, IL-17, and MAPK signaling pathways—were all fully preserved at 2 hours post infection (Supplementary Fig. S2A), while key cascades such as the MAPK and TNF signaling pathways were stably retained at 6 hours post infection (Supplementary Fig. S2B), confirming that the activation of these inflammatory programs is robust across threshold definitions. Given this sustained expression profile and its emergence as a dominant sensor during secondary inflammatory reprogramming, we selected CLEC4E as the primary focus for subsequent functional investigation.

To validate the transcriptomic findings, quantitative real-time PCR was performed in RAW264.7 cells under M2, AM2, M6, and AM6 conditions to assess the expression of downstream inflammatory effectors and the classical M1 polarization marker *Cd86*. At 2 hours post infection, *Nos2*, *Il1b*, and *Clec4e* were significantly upregulated compared with controls (Fig. 3A). By 6 hours post infection, *Nos2*, *Cd86*, *Tnfa*, *Il1b*, *Cxcl1*, and *Clec4e* were robustly and consistently elevated. The prominent and delayed appearance of *Cd86* at 6 hours, following the initial *Clec4e* induction, indicates that early PRR-driven signaling actively progresses toward M1 macrophage phenotype. Western blot analysis further demonstrated increased protein levels of CLEC4E, iNOS, TNF-α, and IL-1β, accompanied by enhanced phosphorylation of p65, particularly at 6 hours post infection (Fig. 3B), indicating the activation of NF-κB signaling.

**Figure 3.**
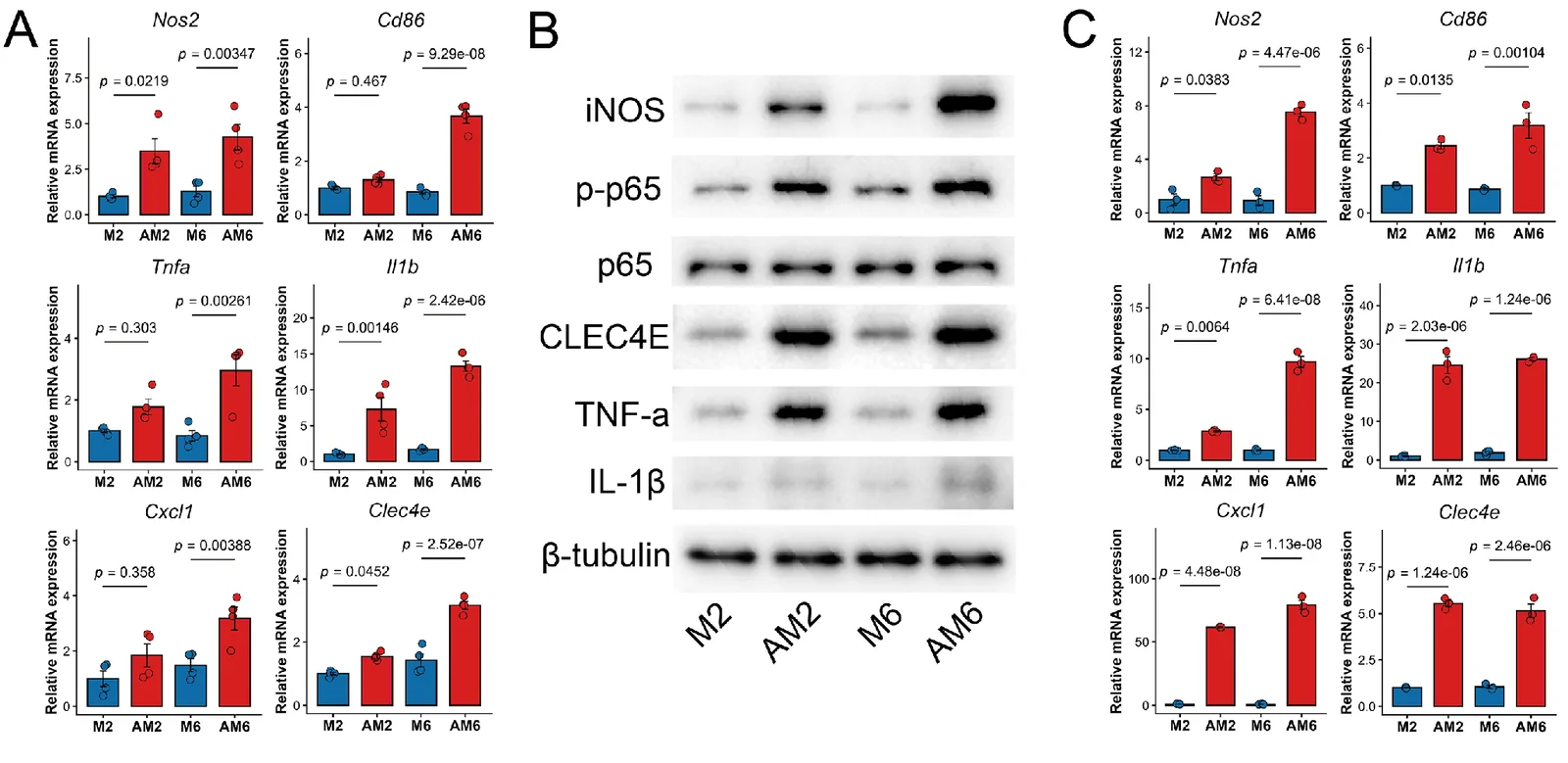
*Acanthamoeba* infection enhances inflammatory responses and Clec4e expression in macrophages. (**A**) Quantitative real-time PCR analysis of inflammatory gene expression in RAW264.7 cells under the M2, AM2, M6, and AM6 conditions (*n* = 4). (**B**) Representative Western blot analysis of iNOS, TNF-α, IL-1β, CLEC4E, and phosphorylated and total p65 in RAW264.7 cells under the indicated conditions. (**C**) Quantitative real-time PCR analysis of inflammatory gene expression in BMDMs under the M2, AM2, M6, and AM6 conditions (*n* = 3). Data are shown as mean ± SEM. Statistical analyses were performed using Welch's *t*-test.

To determine whether this response was reproducible across macrophage systems, BMDMs were examined. Similar to RAW264.7 cells, inflammatory gene expression was more pronounced at 6 hours post infection, including significant induction of *Nos2*, *Cd86*, *Tnfa*, *Il1b*, *Cxcl1*, and *Clec4e* (Fig. 3C). Collectively, these findings demonstrate that *Acanthamoeba* infection induces robust inflammatory activation in macrophages, accompanied by sustained CLEC4E upregulation.

### CLEC4E Promotes Macrophage Inflammatory Responses During *Acanthamoeba* Infection

To examine whether the activation of CLEC4E modulates macrophage responses to *Acanthamoeba* infection, macrophages were pretreated with the CLEC4E agonist TDB or vehicle control (DMSO) for 24 hours before infection. In RAW264.7 cells, TDB pretreatment significantly enhanced the expression of multiple proinflammatory genes after *Acanthamoeba* infection, including *Nos2*, *Cd86*, *Tnfa*, *Il1b*, and *Clec4e*, while *Cxcl1* showed an increasing trend (Fig. 4A). Consistently, immunoblot analysis demonstrated increased protein levels of iNOS, IL-1β, and TNF-α, along with enhanced phosphorylation of p65 in TDB-treated cells compared with controls (Fig. 4B), indicating the potentiation of NF-κB activation.

**Figure 4.**
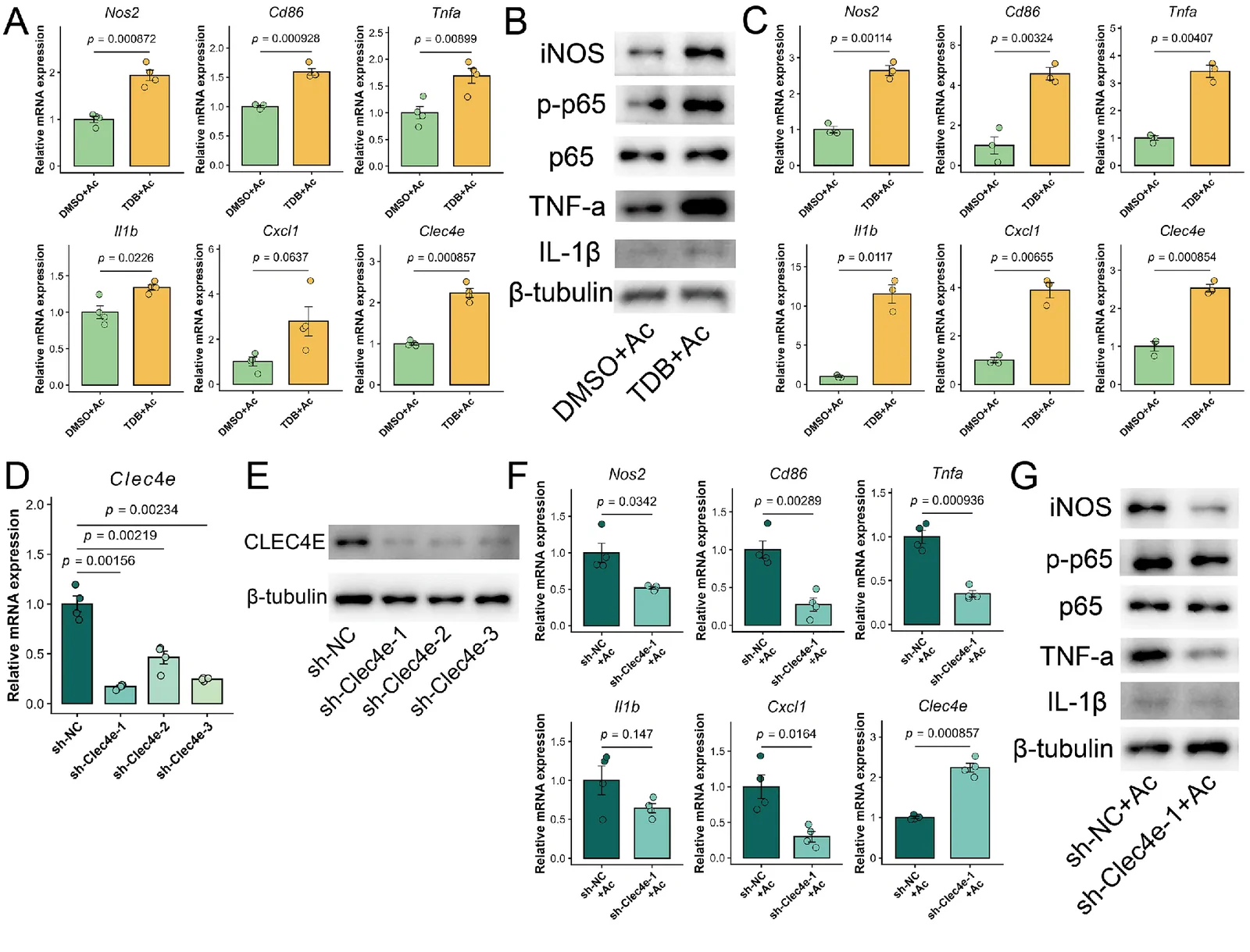
CLEC4E positively regulates macrophage inflammatory responses to *Acanthamoeba* infection. (**A**) Quantitative real-time PCR analysis of inflammatory gene expression in RAW264.7 cells pretreated with DMSO or TDB before infection (*n* = 4). (**B**) Representative Western blot images of iNOS, TNF-α, IL-1β, and phosphorylated and total p65 in RAW264.7 cells under TDB or DMSO pretreatment. (**C**) Quantitative real-time PCR analysis of inflammatory gene expression in BMDMs pretreated with DMSO or TDB (*n* = 3). (**D**) Quantitative real-time PCR validation of Clec4e knockdown efficiency in RAW264.7 cells (*n* = 4). (**E**) Representative Western blot validation of Clec4e knockdown. (**F**) Quantitative real-time PCR analysis of inflammatory gene expression in the nontargeting negative control (sh-NC) and sh-Clec4e-1 groups following infection (*n* = 4). (**G**) Representative Western blot analysis of iNOS, TNF-α, IL-1β, and phosphorylated and total p65 in the sh-NC and sh-Clec4e-1 groups. Data are shown as mean ± SEM. Statistical analyses were performed using Welch's *t*-test.

Similar effects were observed in BMDMs. TDB pretreatment significantly enhanced the expression of multiple proinflammatory genes after *Acanthamoeba* infection, including *Nos2*, *Cd86*, *Tnfa*, *Il1b*, *Cxcl1*, and *Clec4e* (Fig. 4C), supporting the reproducibility of CLEC4E-mediated amplification of inflammatory responses.

To further determine the functional role of CLEC4E, RAW264.7 cells were transduced with shRNA-expressing lentiviruses targeting *Clec4e* before *Acanthamoeba* infection. Quantitative real-time PCR analysis confirmed the efficient knockdown of *Clec4e* expression across multiple shRNA constructs (Fig. 4D), which was further validated at the protein level (Fig. 4E). Among these, sh-Clec4e-1 exhibited the greatest knockdown efficiency and was therefore selected for subsequent functional experiments.

After infection, the sh-Clec4e-1 group showed a significantly decreased expression of inflammatory genes compared with the nontargeting negative control group, including *Nos2*, *Cd86*, *Tnfa*, and *Cxcl1*, whereas *Il1b* showed a decreasing trend without reaching statistical significance (Fig. 4F). Western blot analysis demonstrated reduced iNOS, IL-1β, and TNF-α protein levels, along with diminished phosphorylation of p65 in *Clec4e*-silenced cells (Fig. 4G), indicating impaired NF-κB activation.

### HIF-1α Contributes to CLEC4E-Mediated Inflammatory Amplification During *Acanthamoeba* Infection

To further investigate the mediators associated with CLEC4E, a co-expression analysis was performed using transcriptomic data. The top 50 genes ranked by Pearson correlation coefficient with *Clec4e* were identified (Fig. 5A). Among these genes, *Hif1a* showed a strong positive correlation with *Clec4e*, suggesting a potential functional linkage. GO enrichment analysis of the highly correlated genes revealed significant enrichment in processes, including leukocyte cell–cell adhesion, nucleocytoplasmic transport, and nuclear transport (Fig. 5B), indicating involvement in immune activation and transcriptional regulation. KEGG pathway enrichment analysis of genes highly correlated with *Clec4e* further revealed significant enrichment in multiple inflammation-related signaling pathways. These included the MAPK signaling pathway, ubiquitin-mediated proteolysis, and bacterial infection–associated pathways such as *Salmonella* infection and *Yersinia* infection (Fig. 5C).

**Figure 5.**
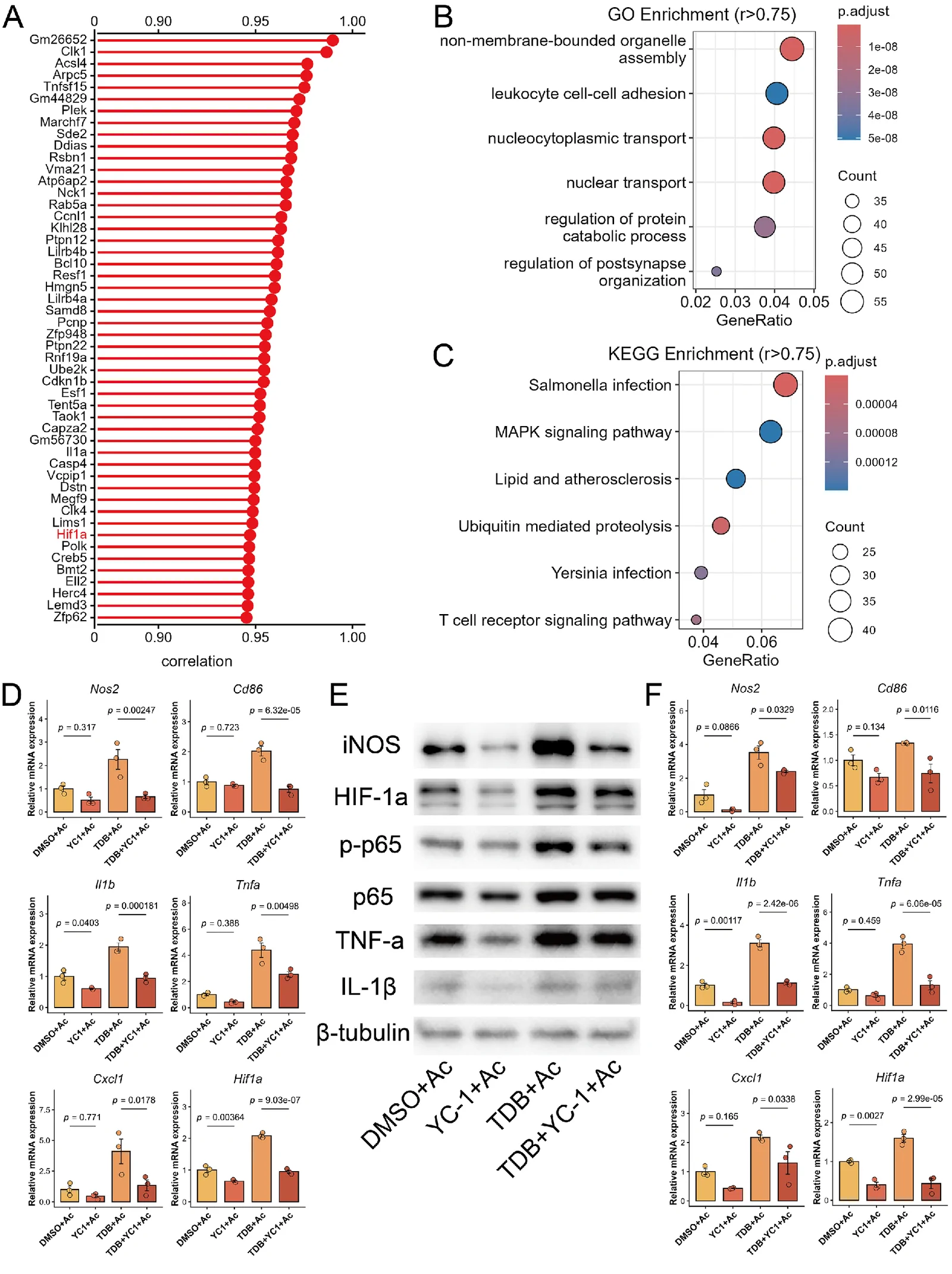
HIF-1α contributes to CLEC4E-mediated inflammatory amplification during *Acanthamoeba* infection. (**A**) Lollipop chart showing the top 50 genes positively correlated with Clec4e expression based on Pearson correlation analysis. (**B**) GO and (**C**) KEGG enrichment analyses of highly correlated genes (*r* > 0.75). (**D**) Quantitative real-time PCR analysis of inflammatory gene expression in RAW264.7 cells treated with DMSO, DMSO+YC-1, TDB, or TDB+YC-1 after infection (*n* = 3). (**E**) Representative Western blot images of iNOS, HIF-1α, TNF-α, IL-1β, and phosphorylated p65 and total p65 in RAW264.7 cells under DMSO, DMSO+YC-1, TDB, or TDB+YC-1 treatment after infection. (**F**) Quantitative real-time PCR analysis of inflammatory gene expressions in BMDMs under the indicated treatment conditions (*n* = 3). Data are shown as mean ± SEM. Statistical analyses were performed using Welch's *t*-test.

To determine whether HIF-1α contributes to infection-induced inflammatory responses, macrophages were treated with the HIF-1α inhibitor YC-1 before *Acanthamoeba* infection. In RAW264.7 cells under basal infection conditions (YC-1 vs. DMSO), YC-1 significantly reduced *Il1b* and *Hif1a* expression, while *Nos2*, *Cd86*, *Tnfa*, and *Cxcl1* did not show significant changes. Under CLEC4E activation conditions (TDB+YC-1 vs. TDB), YC-1 significantly attenuated the TDB-enhanced expression of *Nos2*, *Cd86*, *Il1b*, *Tnfa*, *Cxcl1*, and *Hif1a* (Fig. 5D), indicating that HIF-1α activity is required for the enhanced inflammatory response associated with CLEC4E activation. To further validate the involvement of HIF-1α in CLEC4E-mediated inflammatory amplification, protein expression of key inflammatory mediators was examined by Western blot analysis. Consistent with the transcriptional results, activation of CLEC4E signaling by TDB markedly increased the protein levels of HIF-1α, iNOS, phosphorylated p65, TNF-α, and IL-1β in macrophages (Fig. 5E). The pharmacological inhibition of HIF-1α with YC-1 attenuated the TDB-induced increase in these inflammatory proteins, particularly reducing HIF-1α accumulation and downstream inflammatory mediators.

In BMDMs, a similar pattern was observed. YC-1 reduced *Il1b* and *Hif1a* under basal infection, whereas under CLEC4E activation conditions, the inhibition of HIF-1α significantly suppressed multiple TDB-enhanced inflammatory genes, including *Nos2*, *Cd86*, *Il1b*, and *Hif1a*, with partial effects on *Cxcl1* and *Tnfa* (Fig. 5F). Collectively, these findings suggest that HIF-1α has a limited role in basal macrophage activation and the direct response to *Acanthamoeba* infection but is more prominently involved in the CLEC4E-mediated amplification of inflammatory signaling.

### In Vivo Blockade of CLEC4E Aggravates Experimental AK

To further investigate the role of CLEC4E during AK in vivo, mice were treated with a neutralizing anti-CLEC4E antibody before infection. Slit-lamp examination showed that anti–CLEC4E-treated mice (Mincle Ab group) developed more severe corneal opacity compared with isotype-treated controls (IgG group) (Fig. 6A). Consistently, clinical scores significantly increased in the anti–CLEC4E-treated group at 1, 3, 5, and 7 days post infection (*P* < 0.05) (Fig. 6B). To ensure the validity of the composite clinical score, we examined the intercorrelations among its three components (opacity area, opacity density, and surface regularity) across all observations. The Spearman correlations were modest (ρ = 0.28–0.52), and variance inflation factor values ranged from 1.13 to 1.44, confirming that the three parameters capture distinct aspects of disease severity and can be combined (Supplementary Tables S5 and S6). Furthermore, the relative *Acanthamoeba* burden in the corneal tissues was significantly increased after CLEC4E blockade at day 3 post infection (Fig. 6C).

**Figure 6.**
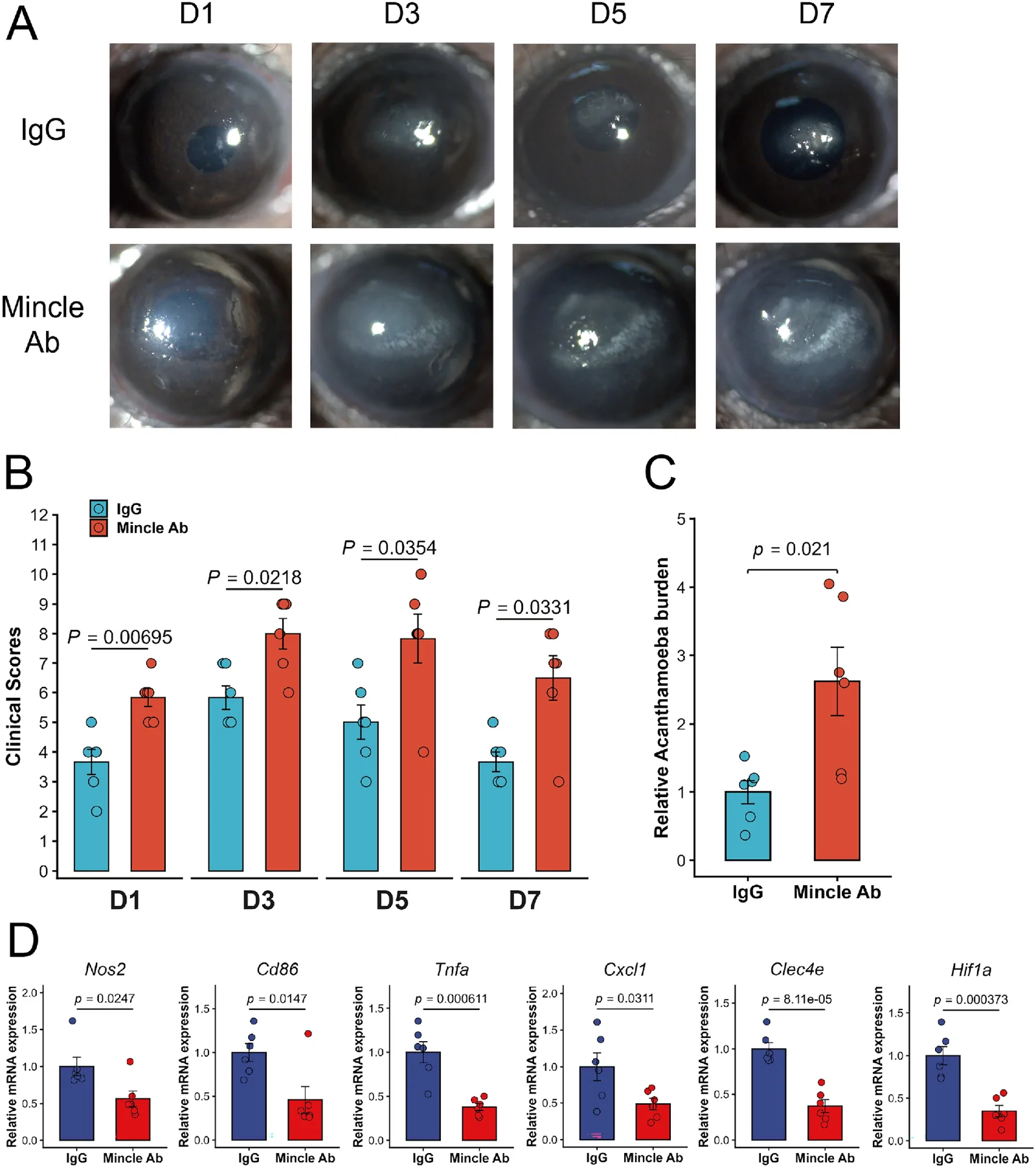
The in vivo blockade of CLEC4E aggravates experimental AK. (**A**) Representative slit-lamp images of corneas from mice treated with control IgG or anti-CLEC4E antibody (Mincle Ab) at 1, 3, 5, and 7 days post infection (D1, D3, D5, and D7, respectively). (**B**) Clinical scores of AK mice at indicated time points (*n* = 6). Clinical scores were compared using the Mann–Whitney *U* test. (**C**) Relative *Acanthamoeba* burden in corneal tissues at D3 post infection (*n* = 6). (**D**) Relative mRNA expression of inflammatory genes in corneal tissues at D1 post infection (*n* = 6). Data are shown as mean ± SEM. Statistical analyses were performed using Welch's *t*-test.

Quantitative real-time PCR analysis revealed a decreased expression of classical inflammatory mediators, including *Nos2*, *Tnfa* and *Cxcl1*, following CLEC4E blockade at day 1 post infection (*P* < 0.05) (Fig. 6D). In addition, the expression of *Hif1a* was also significantly downregulated (*P* < 0.001) (Fig. 6D). Collectively, these findings suggest that CLEC4E contributes to protective innate immune responses during experimental AK, whereas CLEC4E blockade exacerbates corneal opacity and tissue injury in vivo.

## Discussion

This study identifies the CLR CLEC4E as a regulator of macrophage inflammatory responses during *Acanthamoeba* infection. A transcriptomic analysis of human corneal tissues revealed the broad activation of innate immune pathways and increased expression of multiple PRRs in AK corneas, including CLEC4E. In macrophage infection models, *Acanthamoeba* induced sustained CLEC4E expression accompanied by the activation of inflammatory signaling pathways. Functional experiments further demonstrated that the activation of CLEC4E enhanced macrophage inflammatory responses, whereas *Clec4e* knockdown attenuated these responses. In addition, HIF-1α was identified as a mediator contributing to CLEC4E-associated inflammatory amplification. Importantly, the in vivo blockade of CLEC4E aggravated experimental AK, resulting in increased corneal opacity, further supporting a protective role of CLEC4E during infection. Together, these findings suggest that CLEC4E participates in innate immune regulation and macrophage activation during *Acanthamoeba* infection.

CLEC4E is a Syk-coupled CLR predominantly expressed in myeloid cells and functions as a sensor of microbial glycolipids and damage-associated molecular patterns.^18^ Several microbial ligands for CLEC4E have been identified, including trehalose dimycolate from *Mycobacterium tuberculosis*, as well as glycolipid components derived from fungal pathogens such as *Candida*, *Lomentospora*, *Scedosporium*, and *Malassezia.*^24^^–^^26^ Moreover, CLEC4E has been implicated in host responses to intracellular bacteria such as *Orientia* and *Rickettsia.*^19^^,^^27^^,^^28^ In addition to microbial ligands, CLEC4E can also respond to endogenous danger signals such as SAP130 released from necrotic cells, linking tissue damage to inflammatory responses.^20^ In the context of AK, CLEC4E activation may occur through the integration of pathogen-derived components and damage-associated signals released after *Acanthamoeba*-induced epithelial injury. Given that CLEC4E expression was rapidly induced after *Acanthamoeba* exposure, it may contribute to the early amplification of macrophage inflammatory responses. However, the specific ligand responsible for CLEC4E activation during AK remains to be identified.^29^ Recent studies have suggested that *Acanthamoeba*-associated micro-organisms, including intracellular bacteria, may contribute to the complexity of AK pathogenesis.^30^^–^^32^ Therefore, CLEC4E may function together with other PRRs, such as TLR4, to integrate diverse microbial and damage-associated signals and coordinate innate immune responses during infection.^33^^,^^34^ Although CLEC4E may be expressed by multiple immune cell populations, macrophages represent a major CLEC4E-expressing cell type and play central roles in immune activation and inflammatory regulation during infection. Therefore, macrophages were selected as the primary cellular model to investigate the role of CLEC4E signaling in *Acanthamoeba* infection.^18^

The activation of CLEC4E signaling promotes macrophage inflammatory responses through downstream NF-κB and MAPK pathways.^35^^,^^36^ In macrophages, these pathways induce the transcription of proinflammatory cytokines, chemokines such as CXCL1, and antimicrobial effector molecules, including iNOS. While CXCL1 is essential for the rapid recruitment of neutrophils to the site of infection, nitric oxide produced by iNOS can contribute to antimicrobial defense against *Acanthamoeba*, but excessive nitric oxide production may also amplify inflammatory tissue injury.^37^ However, the in vivo outcome is also influenced by the ability of *Acanthamoeba* to withstand host immune pressure. Under adverse or inflammatory conditions, *Acanthamoeba* can differentiate into highly resistant cysts and activate stress-adaptation mechanisms that may facilitate persistence despite macrophage-mediated antimicrobial responses. Thus, enhanced macrophage activation may contribute to pathogen control but may not be sufficient for complete parasite clearance and may simultaneously exacerbate tissue injury.^38^ Therefore, iNOS-derived responses may represent a balance between pathogen control and inflammation-associated tissue damage during AK.^39^^,^^40^ Consistent with these mechanisms, our study demonstrated the robust induction of *Nos2*, *Il1b*, *Tnfa*, and *Cxcl1* after *Acanthamoeba* infection. The parallel sustained upregulation of *Clec4e* and *Nlrp3* at 6 hours post infection, coupled with the robust induction of IL-1β across both time points, suggests a potential mechanistic convergence on inflammasome activation. It is plausible that CLEC4E signaling potentiates NLRP3 inflammasome function during *Acanthamoeba* infection, representing an intriguing axis for future investigation. Using TDB, a synthetic CLEC4E agonist, we further demonstrated that enhanced CLEC4E signaling potentiated these inflammatory responses, whereas *Clec4e* knockdown significantly attenuated them, supporting a functional role for CLEC4E in macrophage activation. Notably, the in vivo neutralization of CLEC4E exacerbated corneal inflammation and disease severity during experimental AK, indicating that inflammatory activation promoted by CLEC4E is required for effective innate immune defense during infection. Therefore, CLEC4E-mediated signaling may contribute to the host defense against *Acanthamoeba* by amplifying macrophage antimicrobial functions and inflammatory signaling.

HIF-1α is a key regulator of macrophage inflammatory responses and metabolic reprogramming, supporting glycolytic metabolism and the production of proinflammatory mediators.^41^^–^^43^ In our study, the inhibition of HIF-1α selectively attenuated the CLEC4E-enhanced expression of inflammatory genes and proteins, while having limited effects on baseline responses to *Acanthamoeba* infection, indicating that HIF-1α is specifically required for the CLEC4E-mediated amplification of macrophage activation. These results suggest that CLEC4E activation is associated with HIF-1α–dependent inflammatory and metabolic programs during infection. Together, these findings highlight HIF-1α as a mediator of the CLEC4E-driven amplification of macrophage responses during *Acanthamoeba* infection.

Several limitations of the present study should be acknowledged. First, although CLEC4E was identified as an important regulator of macrophage responses during *Acanthamoeba* infection, the specific ligand recognized by CLEC4E in this context remains unknown. Although TDB was used as a synthetic CLEC4E agonist to evaluate the functional role of CLEC4E signaling, it does not represent an *Acanthamoeba*-derived ligand. Second, although our results indicate that HIF-1α contributes to CLEC4E-mediated inflammatory amplification, the precise molecular mechanism linking CLEC4E activation to HIF-1α regulation was not investigated in detail. Although HIF-1α is known to regulate metabolic reprogramming in macrophages, the relationship between CLEC4E signaling, HIF-1α activation, and metabolic changes during *Acanthamoeba* infection was not examined in the present study. Third, only female mice were used in this study, and the potential influence of sex as a biological variable was not evaluated. Fourth, although macrophages are important contributors to innate immune responses in AK, the unique immune microenvironment of the cornea and other immune populations may also influence disease progression. The interactions between CLEC4E-expressing macrophages and other immune cell subsets were not investigated in this study and require further exploration. Fifth, because macrophages are professional phagocytes, the mechanical engulfment of large *Acanthamoeba* trophozoites may inherently trigger nonspecific activation pathways. Therefore, the early transcriptomic reprogramming observed likely reflects a combination of basal phagocytic stress and specific PRR-mediated signaling. Disentangling the exact contribution of a specific receptor activation from general phagocytosis will warrant further investigation in future studies by comparing these responses with the engulfment of inert particles of a similar size. Future studies addressing these questions will further clarify the mechanisms underlying innate immune recognition and macrophage activation in AK.

In summary, the present study demonstrated that CLEC4E functions as a key regulator of macrophage inflammatory responses during *Acanthamoeba* infection, and that HIF-1α mediates the CLEC4E-dependent amplification of these responses. CLEC4E signaling enhances macrophage antimicrobial and inflammatory functions through HIF-1α–dependent pathways, highlighting its central role in innate immune recognition in the cornea. These findings provide new insights into the molecular mechanisms of host defense in AK and suggest that targeting CLEC4E–HIF-1α signaling could represent a potential strategy to modulate corneal inflammation and improve disease outcomes.

## Supplementary Material

Supplement 1
