# Supplementary material for: CLEC4E-Mediated Macrophage Activation Contributes to Host Defense in Acanthamoeba Keratitis
Source: Invest Ophthalmol Vis Sci. 2026 Oct 26;67(12):63. doi: 10.1167/iovs.67.12.63 (PMC13722982; doi:10.1167/iovs.67.12.63)
Supplement: Supplementary file 1 [file iovs-67-12-63_s001.docx]

Supplementary Table S1. Visual Scoring Criteria for Murine *Acanthamoeba* Keratitis

| Parameter | Grade 0 | Grade 1 | Grade 2 | Grade 3 | Grade 4 |
| --- | --- | --- | --- | --- | --- |
| Corneal opacity area | No corneal opacity | Opacity involving 1%–25% of the corneal area | Opacity involving 26%–50% of the corneal area | Opacity involving 51%–75% of the corneal area | Opacity involving 76%–100% of the corneal area |
| Opacity density | No corneal opacity | Slight cloudiness; outline of iris and pupil remains clearly discernible | Cloudy cornea; outline of iris and pupil remains visible | Dense but non-uniform opacity; iris and pupil structures are difficult to distinguish | Uniform dense opacity; iris and pupil structures are not visible |
| **Surface regularity** | Normal, smooth corneal surface | Slight surface irregularity | Rough corneal surface with mild swelling | Significant swelling with crater formation or severe descemetocele formation | Corneal perforation |

Supplementary Table S2. Primers used in this study

| Primer name | Forward | Reverse |
| --- | --- | --- |
| Mouse |  |  |
| Ppia | GAGCTGTTTGCAGACAAAGTTC | CCCTGGCACATGAATCCTGG |
| Nos2 | CCCTTCAATGGTTGGTACATGG | ACATTGATCTCCGTGACAGCC |
| Cd86 | CTGCTCATCATTGTATGTCAC | ACTGCCTTCACTCTGCATTTG |
| Tnfa | ACCCTCACACTCAGATCATCTT | GGTTGTCTTTGAGATCCATGC |
| Il1b | TTCAGGCAGCAGTATCACTC | GAAGGTCCACGGGAAGACAC |
| Cxcl1 | GACTCCAGCCACACTCCAAC | TGACAGCGCAGCTCATTG |
| Clec4e | CATCCCACCACACAGAGAGA | GGTGATGAAACAGCCACTGA |
| Hif1a | CCCATTCCTCATCCGTCAAATA | CCTGTGGTGACTTGTCCTTTAG |

Supplementary Table S3. Expression profiles of previously reported immune genes in human AK corneas

| Gene | log2FoldChange | pvalue | padj |
| --- | --- | --- | --- |
| CD8A | 5.03 | <0.001 | <0.001 |
| CD14 | 2.61 | <0.001 | 0.003 |
| CD33 | 5.35 | <0.001 | <0.001 |
| SLAMF7 | 7.88 | <0.001 | <0.001 |
| STAT6 | 0.83 | 0.030 | 0.113 |
| CCL3 | 3.53 | <0.001 | 0.002 |
| CCL23 | 6.87 | 0.004 | 0.023 |

Supplementary Table S4. Previously reported top 30 enriched KEGG pathways in human AK corneas

| Cytokine-cytokine receptor interaction | Chemokine signaling pathway | Natural killer cell mediated cytotoxicity |
| --- | --- | --- |
| Hematopoietic cell lineage | Type I diabetes mellitus | Tuberculosis |
| Staphylococcus aureus infection | Antigen processing and presentation | Viral myocarditis |
| Viral protein interaction with cytokine and cytokine receptor | Osteoclast differentiation | Asthma |
| Graft-versus-host disease | Th17 cell differentiation | Epstein-Barr virus infection |
| Allograft rejection | Intestinal immune network for IgA production | Malaria |
| Cell adhesion molecules | Th1 and Th2 cell differentiation | B cell receptor signaling pathway |
| Rheumatoid arthritis | Phagosome | JAK-STAT signaling pathway |
| Leishmaniasis | Autoimmune thyroid disease | African trypanosomiasis |
| Inflammatory bowel disease | Primary immunodeficiency | NOD-like receptor signaling pathway |

Supplementary Figure S1. (A) GO enrichment and (B) KEGG pathway enrichment analysis of upregulated genes in AK corneas under a threshold of log2FC ≥ 1.0 and adjusted P < 0.05.

Supplementary Figure S2. KEGG pathway enrichment analysis of upregulated genes at (A) 2 h and (B) 6 h post-infection under a threshold of log2FC ≥ 1.0 and adjusted P < 0.05.

Supplementary Table S5. Intercorrelations of clinical score components using Spearman's correlation coefficients (ρ)

| Comparison | ρ | *P* value | ρ² |
| --- | --- | --- | --- |
| Area vs Density | 0.524 | 0.0001 | 0.274 |
| Area vs Surface | 0.309 | 0.0327 | 0.095 |
| Density vs Surface | 0.278 | 0.0558 | 0.077 |

Supplementary Table S6. Variance inflation factors of clinical score components

| Component | VIF |
| --- | --- |
| Area | 1.435 |
| Density | 1.407 |
| Surface | 1.129 |
